# Computational prediction of hinge axes in proteins

**DOI:** 10.1186/1471-2105-15-S8-S2

**Published:** 2014-07-14

**Authors:** Rittika Shamsuddin, Milka Doktorova, Sheila Jaswal, Audrey Lee-St John, Kathryn McMenimen

**Affiliations:** 1 Computer Science, University of Texas, Dallas, TX, USA; 2 Tri-Institutional Training Program in Computational Biology and Medicine, Weill Cornell Medical College, New York, NY, USA; 3 Dept. of Chemistry, Amherst College, Amherst, MA, USA; 4 Dept. of Computer Science and Dept. of Chemistry, Mount Holyoke College, South Hadley, MA, USA

**Keywords:** protein flexibility, rigidity theory, linear algebra

## Abstract

**Background:**

A protein's function is determined by the wide range of motions exhibited by its 3D structure. However, current experimental techniques are not able to reliably provide the level of detail required for elucidating the exact mechanisms of protein motion essential for effective drug screening and design. Computational tools are instrumental in the study of the underlying structure-function relationship. We focus on a special type of proteins called "hinge proteins" which exhibit a motion that can be interpreted as a rotation of one domain relative to another.

**Results:**

This work proposes a computational approach that uses the geometric structure of a single conformation to predict the feasible motions of the protein and is founded in recent work from *rigidity theory*, an area of mathematics that studies flexibility properties of general structures. Given a single conformational state, our analysis predicts a relative *axis of motion *between two specified domains. We analyze a dataset of 19 structures known to exhibit this hinge-like behavior. For 15, the predicted axis is consistent with a motion to a second, known conformation. We present a detailed case study for three proteins whose dynamics have been well-studied in the literature: calmodulin, the LAO binding protein and the Bence-Jones protein.

**Conclusions:**

Our results show that incorporating rigidity-theoretic analyses can lead to effective computational methods for understanding hinge motions in macromolecules. This initial investigation is the first step towards a new tool for probing the structure-dynamics relationship in proteins.

## Background

Proteins play a significant role in virtually all biological processes. These macromolecules are composed of sequences of amino acids folded into 3D shapes of varying size and complexity. The structures of many proteins have been determined experimentally and are easily accessible [1]. The key to protein function, however, is the wide range of motions exhibited by the molecules, from local vibrational fluctuations to larger global movements significantly altering the conformational state [2]. The motions of biological interest occur on the timescales of picoseconds to nanoseconds, which makes their study challenging. Only a few experimental techniques, such as NMR and single-molecule FRET, are capable of probing dynamics at this level [3-6]. However, these techniques are not able to reliably provide the level of detail required for elucidating the exact mechanisms of protein motion and the underlying structure-function relationship, essential for effective drug screening and design. Theoretical models and computational tools are instrumental for gaining better mechanistic understanding and predictive power.

**Figure 1:**
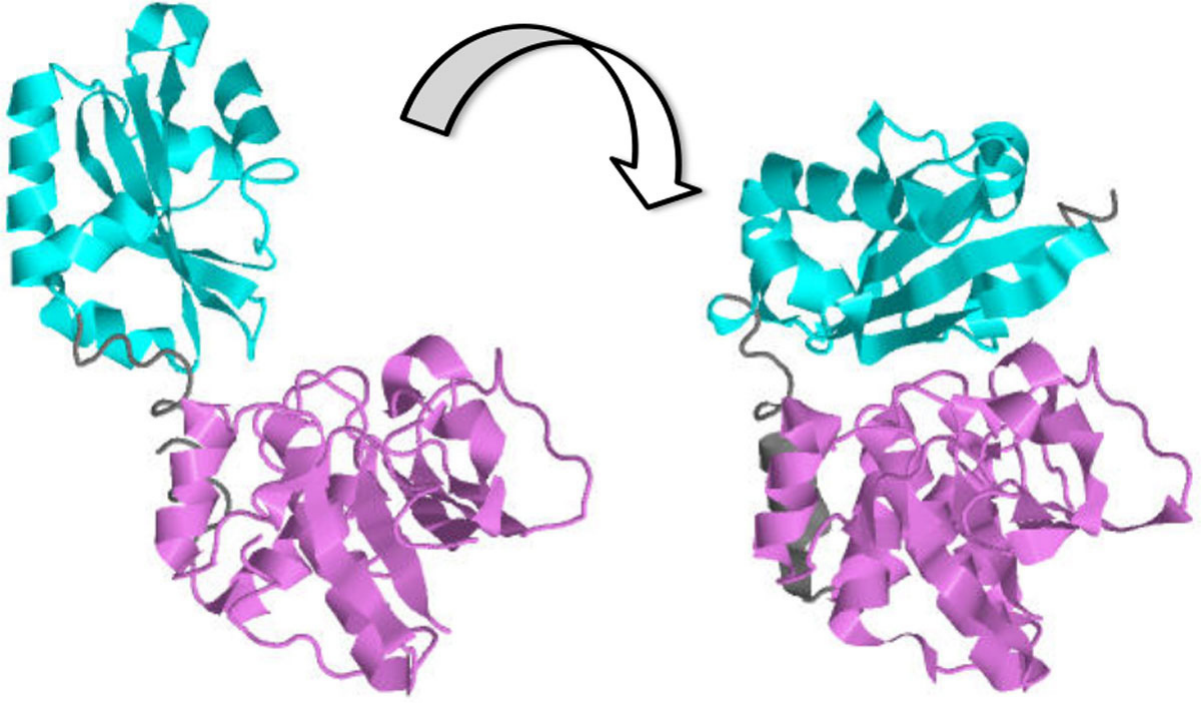
**An example of a "hinge" protein: a conformational change from the *open *state of inorganic pyrophosphatase [PDB:1K23] to the *closed *state [PDB:1K20] causes one domain to rotate towards the other**.

In this work, we demonstrate the applicability of rigidity theory, an area of mathematics that studies the flexibility properties of general structures, to the analysis of protein dynamics. In particular, we focus on a set of proteins that exhibit "hinge" behavior, a rotational movement of one domain of the protein relative to another (see Figure 1). Recent work [7] presented an approach to identifying revolute (allowing a single rotational motion) and prismatic (allowing a single translational motion) joints in Computer Aided Design structures; hinge proteins exhibit behavior very similar to that allowed by revolute joints. By analyzing a protein's geometric structure from this perspective, we can predict the relative axis of motion for a given pair of domains, providing a quantitative description of the molecule's range of motion. The success of our approach demonstrates that rigidity theory is a powerful tool which can be used to understand the geometric properties determining the dynamics of macromolecules. To the best of our knowledge, this is the first computational method to predict such an axis based on a *single conformational state*.

### Related work

Computational methods for predicting hinges in proteins generally focus on determining which residues comprise the "hinge" joint, expected to allow flexibility that results in a motion of two larger domains. The most closely related approaches include Stonehinge [8], HingeProt [9], and DynDom [10]. Both Stonehinge and HingeProt rely on analysis of rigidity and flexibility properties of the protein by using elastic network models; Stonehinge additionally incorporates the same underlying rigidity theory as KINARI [11] to find a cluster decomposition. These methods seek to pinpoint the location of the "hinge" joint; while this is done from a single conformation as input, a predicted axis of motion is not part of the output. The approach of DynDom does identify an axis of motion, but requires two conformations as input.

### Contributions

We present a computational approach for predicting the type of motion allowed by a protein; as input, we require a single structure with two domains identified for which relative motion should be studied. Our analysis models the protein as a geometric structure studied in rigidity theory and predicts the relative axis of motion. We use KINARI [11] to perform initial rigidity analysis, resulting in a decomposition of the structure into rigid regions, or "clusters." This reduces the complexity of the protein, allowing subsequent computational analysis for predicting the *axis of motion*. We take steric hindrance, a molecular property not modeled by the theory, into account by incorporating Rosetta energy calculations [12] when sampling conformations near the native state. We evaluate our approach on 19 structures of proteins known to exhibit hinge-like motions and verify that the predicted axis of motion is consistent with a second conformation for 15 of them. To illustrate our results, we present a case study of three of the proteins from our dataset: calmodulin, the LAO binding protein and the Bence-Jones protein.

## Methodology

Our approach is based on results from infinitesimal rigidity theory. We begin with a brief overview of the relevant theoretical concepts, then present our analysis pipeline. For a more thorough treatment of classical rigidity theory, see [13]; for further explanation of the theory behind identifying revolute and prismatic joints, see [7].

### Preliminaries

**Figure 2:**
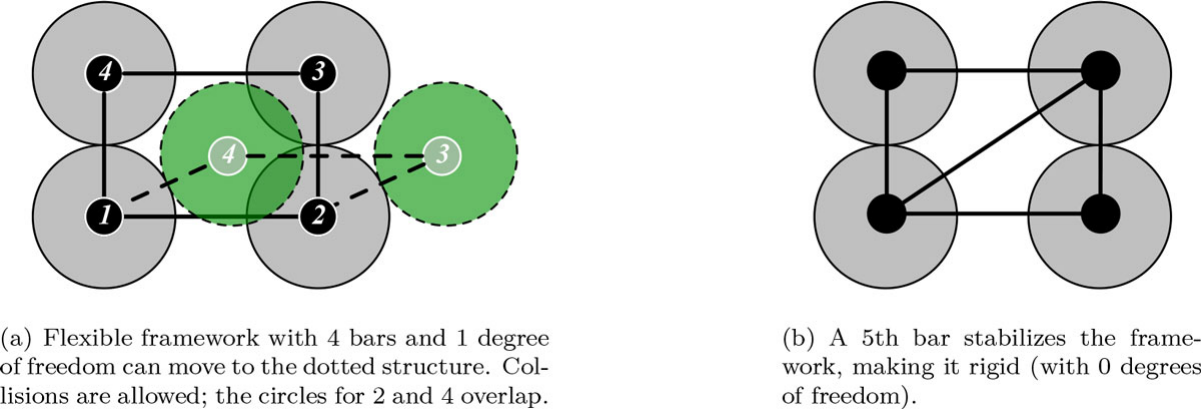
**A structure on 4 circles with distance constraints in the plane can be modeled as a bar-and-joint structure with universal joints placed at the center of each circle and fixed-length bars between them**. (a) Flexible framework with 4 bars and 1 degree of freedom can move to the dotted structure. Collisions are allowed; the circles for 2 and 4 overlap. (b) A 5th bar stabilizes the framework, making it rigid (with 0 degrees of freedom).

Rigidity theory considers geometric constraint structures, such as the classical *bar-and-joint framework*. A bar-and-joint framework consists of universal joints whose motion is constrained by fixed-length bars and can be expressed by an algebraic system of quadratic distance equations. See Figure 2 for example bar-and-joint frameworks with 4 joints.

#### Infinitesimal rigidity theory

**Figure 3:**
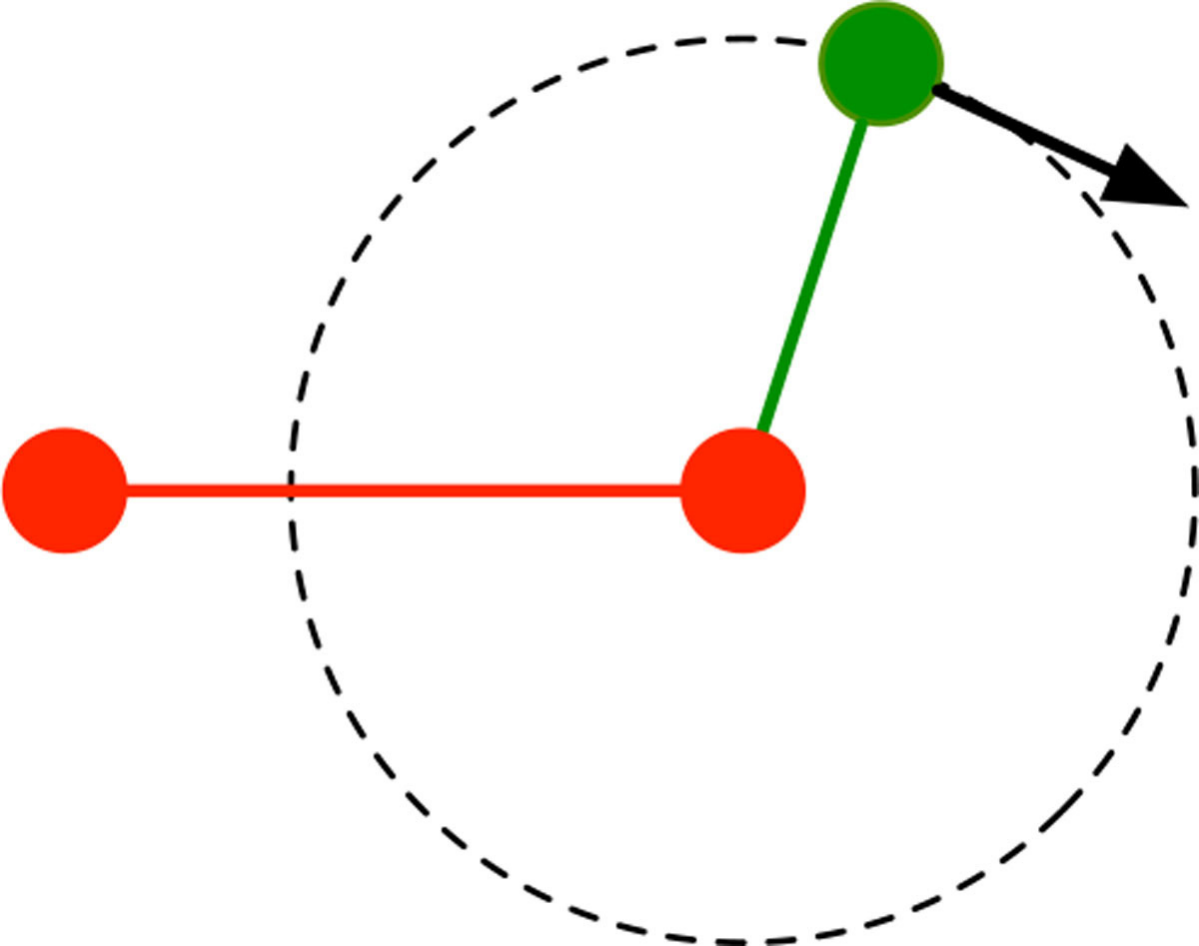
**Infinitesimal rigidity theory of a bar-and-joint framework in the plane: pinning the red bar, the black velocity vector maintains the length of the green bar infinitesimally**. It is tangent to the dotted circle whose radius is defined by the bar's length.

*Infinitesimal rigidity theory *studies the first-order behavior of the system of quadratic distance equations, and a **rigidity matrix **encodes the corresponding linear constraints. The null space of the rigidity matrix determines the *infinitesimal motion space *(for brevity, we will omit "infinitesimal" for the remainder of this paper), which assigns a velocity vector to each joint such that the bars maintain their lengths infinitesimally; refer to Figure 3. Since the trivial instantaneous rigid body motions (in the plane, translation in the *x*- and *y*-directions and rotation about the origin) are always contained in the motion space, additional "pinning" rows are often added to the rigidity matrix so that the null space contains only internal motions.

Assuming the framework is not in a singular position, the dimension of the motion space after pinning defines the number of *degrees of freedom *available to the framework; this is equivalent to the minimum number of bars whose addition would stabilize the framework. For example, the 4-bar mechanism in Figure 2(a) has 1 degree of freedom, as the addition of a single bar creates a rigid structure (Figure 2(b)). In 2D, *generic *rigidity of a bar-and-joint structure is characterized by a graph-theoretic property proven by Laman [14]; however, in 3D, no analogous result is known. (Intuitively, the term "generic" indicates that the structure is not in a "special position" - the technical definition of genericity is outside the scope of this paper.) For 3D *body-bar-hinge frameworks*, composed of rigid bodies with fixed-length bars or hinges between them, a similar graph-theoretic characterization is given by Tay [15,16]. A bar imposes a distance constraint between two points on the respective bodies, and a hinge allows only a rotational degree of freedom.

The KINARI software that we use models a protein as a body-bar-hinge structure by assigning bars or hinges to chemical interactions computed to be present in the protein; for example, a covalent bond is modeled as a hinge, allowing only the dihedral angle to vary [11]. The infinitesimal rigidity theory of body-bar-hinge structures is analogous to that of bar-and-joint structures: a rigidity matrix encodes the first-order behavior of the constraints, and its null space gives the motion space.

#### Instantaneous motions of body-bar-hinge frameworks

Since we use the motion space of a body-bar-hinge framework in our analysis, we now provide a few more details about instantaneous motions in 3D relevant to this work.

By Chasles' Theorem, every rigid body motion in 3D can be described by a *screw *motion: a rotation and translation along a *screw axis*. One can imagine a screw motion as being analogous to traveling along an alpha helix, with the screw axis defined by the helix's direction and placement. As a consequence, every instantaneous rigid body motion can be described by a *twist*: an instantaneous rotation and translation along a *twist axis*.

We represent a twist with a 6-vector (*ω*_1_*, ω*_2_*, ω*_3_*, v*_1_*, v*_2_*, v*_3_), which can be further decomposed into two vectors of length 3: *ω *= (*ω*_1_*, ω*_2_*, ω*_3_) and **v **= (*v*_1_*, v*_2_*, v*_3_). The vector *ω *is the *angular velocity*, giving the direction of the twist axis and speed of rotation about it (via its magnitude). The remaining translational speed and position of the twist axis in 3D can be decoded from the vector **v**. When 〈*ω*, **v**〉 = 0, where 〈 〉 denotes the dot product, the twist satisfies a relation called the *Plücker relation *and corresponds to an instantaneous motion that is either a pure rotation or a pure translation. Given a twist (*ω*, **v**) for a rigid body, we can compute the instantaneous velocity **p' **for a point **p **on the body according to the following formula (see, e.g., [17], page 43):

p′=ω×p+v

For a body-bar-hinge framework with *n *bodies, a motion of the whole structure assigns a twist to each body and can be described by a vector of length 6*n*. The motion space for the framework, which is a vector space of dimension *d*, may be described by a set of *d *basis vectors **b**_1_*, . . . *, **b***_d_*; a motion vector **s **in the space can be expressed as a linear combination of the basis vectors: $s=\overset{d}{\underset{i=1}{\sum }}c_{i}{\text{b}}_{i}$ for some set of "weight" coefficients *c*_1_*, . . . , c_d _*ϵ ℝ.

### Approach

**Figure 4:**
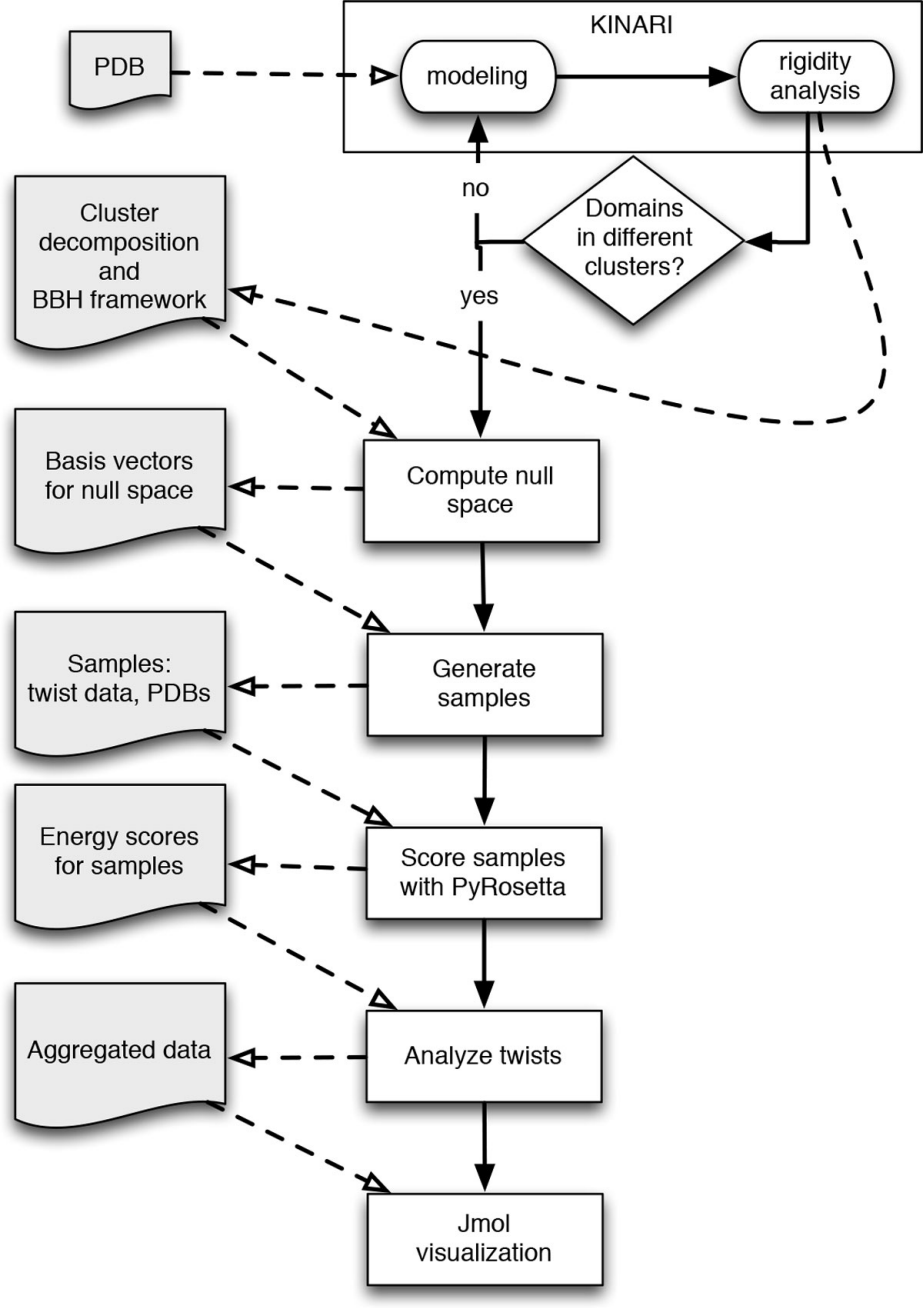
**An overview of the steps used in our analysis**. The left side (shaded, connected with dotted arrows) denotes data.

For the analysis, we picked a set of 19 structures of hinge proteins previously analyzed by Stonehinge [8]. Table 1 provides a summary of the dataset. Figure 4 presents an overview of our approach for analyzing each structure.

**Table 1:** Analyzed protein dataset.

Protein	PDB ID	KINARI cutoff	Pinned cluster	Moving cluster	Twist purity
**Calmodulin (calcium-free)**	1CFD	default	0	1	77.1269
**Calmodulin (Ca**2+ **-bound; open)**	1CLL	-2	0	1	95.435
**Calmodulin (Ca2+ -bound; closed)**	2BBM(A)	default	4	7	150.339
**LAO binding protein (open)**	1LST	default	0	1	91.5669
**LAO binding protein (closed)**	2LAO	default	0	1	98.1407
Bence-Jones protein (open)	4BJL(B)	-1.25	0	1	97.0153
**Bence-Jones protein (closed)**	4BJL(A)	-1.25	0	1	99.0822
**cAMP-dependent protein kinase (open)**	1CTP	-1.9	0	2	89.7223
**cAMP-dependent protein kinase (closed)**	1ATP	-1.9	0	1	93.2537
**Adenylate kinase (open)**	2AK3(A)	default	0	1	95.2801
Adenylate kinase (closed)	1AKE(A)	-2.5	1	0	91.4916
**Glutamine binding protein (open)**	1GGG(A)	default	1	0	105.321
**Glutamine binding protein (closed)**	1WDN	-2	0	1	90
**DNA polymerase *β *(open)**	2BPG(A)	default	0	1	109.657
DNA polymerase *β *(closed)	1BPD	default	0	1	86.6385
**Inorganic pyrophosphatase (open)**	1K23(A)	default	0	1	91.6584
**Inorganic pyrophosphatase (closed)**	1K20(A)	-3	1	0	80.6779
**Ribose binding protein (open)**	1URP(C)	-2	0	1	75.4811
Ribose binding protein (closed)	2DRI	-2.65	1	0	89.4672

For structures where the PDB contains more than one chain, the analyzed chain is indicated within parentheses. The KINARI cutoff column lists the value of the energy cutoff used for including hydrogen bonds. Bold entries indicate structures for which predicted axis of motion is consistent with second conformation.

#### Rigid cluster decomposition with KINARI

We use the KINARI-Web application [11] to model each protein structure as an initial set of bodies (generally one per atom) with constraints between them (determined by inter-atomic chemical interactions). Depending on the nature of the interactions, KINARI represents them as bars or hinges allowing certain degrees of freedom; these choices are adjustable parameters and can thus be modified. Once set, the software analyzes the rigidity of the structure and produces a *cluster decomposition *that reduces the complexity of the initial body-bar-hinge model, where bodies in the original model are grouped into larger rigid clusters.

We only adjust the parameter for hydrogen bonds, which are calculated by KINARI based on the geometry of the structure and are assigned an energy value denoting their strength (the smaller the energy, the stronger the bond). By default, all hydrogen bonds, including the weakest ones, are modeled as hinges with a single degree of freedom. However, this representation may overly restrict the motion of the structure by producing very large rigid clusters where the domains of interest are grouped into the same cluster. In this case, we adjust the hydrogen bond energy cutoff parameter to remove the weakest hydrogen bonds from the model (preserving the default modeling of a hydrogen bond as a hinge). The rigidity analysis is then performed again to produce a new cluster decomposition. This process is repeated until an energy value is found that produces a cluster decomposition with the two domains in distinct clusters.

We proceed with the analysis using the corresponding body-bar-hinge (BBH) framework output by KINARI: each cluster is itself a rigid body, connected to other clusters with bars and hinges. The clusters are labeled by size, with Cluster 0 containing the largest number of atoms. To maintain consistency, we will refer to rigid bodies as "clusters" for the remainder of this paper.

#### Motion space calculation

We choose two clusters to represent the domains whose relevant motion we are studying. We pin one and refer to it as the **pinned cluster**; the other is called the **moving cluster**. Let *n *be the number of clusters and *d *the number of degrees of freedom of the BBH framework output by KINARI. We create the rigidity matrix for the BBH framework, adding the appropriate rows to eliminate motion of the **pinned cluster**, and compute its null space, or motion space. The motion space is output as a set of *d *basis vectors **b***i*, where *i *= 1*, . . . , d*. This is used to generate 1000 **samples**, where each sample is created by the following process:

1. Randomly generate *d *weight coefficients between 0 and 1: *c_i _*for *i *= 1*, . . . , d*.

2. Compute the resulting linear combination of basis vectors: $s=\overset{d}{\underset{i=1}{\sum }}c_{i}{\text{b}}_{i}$

3. Interpret **s **= (**t**_1_*, . . . *, **t***_n_*), where each **t***_i _*ϵ ℝ^6^. Let each **t***_i _*= (*ω_i_*, **v***_i_*), where *ω_i_*, **v***_i _*ϵ ℝ^3^.

For each cluster *i*, let {**p**_1_*, . . . *, **p***_ni_*} be the set of positions of the *n_i _*atoms found in the cluster. For each atom position **p***_j_*, compute **p**'*_j _*= *ω_i _× ***p***_j _*+ **v***_i _*and move the atom in the direction **p**'*_j _*to compute its new position.

4. Output **s **(twists for each cluster) and a PDB with the updated positions.

#### Steric hindrance

Rigidity theory does not consider collisions (see Figure 2(a)), as it is based on a system of linear *equations*; collisions would require *inequality *constraints. However, due to the close packing of atoms in a protein, steric hindrance plays a significant role in the allowable conformations near the native state. We generate the samples by moving each atom an "infinitesimal" distance using the computed motion space, but the direction of many of these motions may be biologically infeasible. Therefore, we use PyRosetta [12,18] to calculate the energy of each generated PDB structure and determine how favorable the motion is. Note that we do not relax the structure, but instead use the computed score to select the most appropriate set of motions for further analysis.

**Figure 5:**
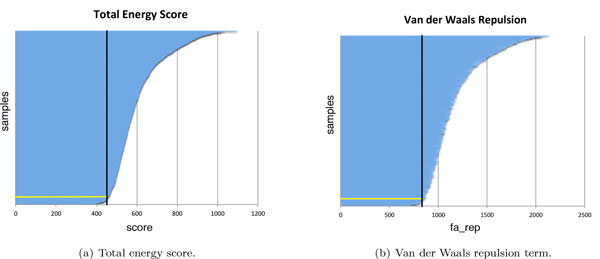
**Rosetta scores computed with PyRosetta for 1000 samples of apo calmodulin [PDB:1CFD]; each sample is represented by a (very thin) horizontal bar, whose length corresponds to its score**. The bars are sorted vertically in descending order by the total energy score. The yellow horizontal line denotes the cutoff of 5% of the samples, with the black vertical line denoting the corresponding score. (a) Total energy score. (b) Van der Waals repulsion term.

To illustrate the treatment of a typical protein from our dataset, we present the results for calcium-free calmodulin [PDB:1CFD] in Figure 5 with each sample represented as a (very thin) horizontal bar. The total energy score is shown in Figure 5(a), and the Van der Waals repulsion term (denoted fa_rep in the Rosetta scoring function) [12] is shown in Figure 5(b); the 1000 samples are sorted in descending order by the total energy score. We found the fa_rep term to be the only term to vary significantly across the samples. This behavior is consistent with our hypothesis that collisions between the atoms increase the Van der Waals repulsion between them, confirming that steric hindrance must be taken into consideration. We therefore restrict further analysis to the most biologically feasible samples, working with lowest 5% in terms of total energy scores.

#### Twist analysis and aggregated data

For each twist (*ω*, **v**), we compute the angle *α *(in degrees) between the two 3-vectors *ω *and **v **using the dot product 〈*ω*, **v**〉; *α *can take on values ranging from 0*° *to 180*°*. Recall that the twist is a pure rotation or translation if 〈*ω*, **v**〉 = 0: if **v **is the zero vector, then the twist is a *pure rotation *about a line through the origin with direction *ω*; if *ω *is the zero vector, then the twist is a *pure translation *in the direction of **v**. Therefore, we refer to the computed angle *α *as the *twist purity*; a value of *α *close to 90*° *corresponds to a dot product close to 0. Values further from 90*° *correspond to more general *screw *motions (with both rotational and translational components).

We aggregate data over the twists used to generate the lowest energy samples, and compute the mean twist and the mean twist purity for the **moving cluster**. From the mean twist, we extract the twist axis, referring to it as the average *axis of motion*. This axis, as well as the corresponding twist purity, give a quantitative description of the motion of the moving cluster relative to the pinned cluster.

## Results and discussion

Refer to Tables 1 and 2 for a summary of our results. For each structure in the dataset, we evaluate the validity of our predictions by manually comparing with a second conformation. We first align the two conformations on the pinned cluster using PyMol, then generate a Jmol script to display the average axis of motion. By visually comparing the conformations in the 3D viewer, we determine if the computed axis is consistent with a motion that allows a feasible pathway for the moving cluster's two positions. For 15 of the structures, the computed axis was consistent with a motion between the analyzed structure and a second conformation; these are listed as **bold **entries in the tables.

**Table 2:** Aggregated twist data.

PDB ID	Mean twist	Pair of points on mean twist axis	
**1CFD**	(6.5286,-5.7547,3.9852,-1.6767,-2.3933,1)	(-0.0413,0.1442,0.2759)	(6.4873,-5.6105,4.2610)
**1CLL**	(7.7431,-7.9801,-8.0737,1.2118,1.0044,1)	(-0.0007,0.0928,-0.0924)	(7.7424,-7.8873,-8.1661)
**2BBM(A)**	(-1.8069,-0.4130,-2.3290,0.2820,0.0460,1)	(0.0345,-0.1298,-0.0038)	(-1.7723,-0.5428,-2.3328)
**1LST**	(118.5740,-78.1629,34.8737,-1.3842,-1.4945,1)	(0.0012,0.0078,0.0133)	(118.5752,-78.1551,34.8870)
**2LAO**	(-82.1258,3.2921,25.4673,1.6032,3.1679,1)	(0.0105,-0.0166,0.0359)	(-82.1153,3.2755,25.5032)
4BJL(B)	(-46.0200,25.3217,-16.3867,0.4717,1.2093,1)	(-0.0149,-0.0126,0.0223)	(-46.0349,25.3091,-16.3644)
**4BJL(A)**	(106.0130,32.4798,-8.2077,0.1640,-1.7414,1)	(-0.0015,0.0087,0.0154)	(106.0115,32.4885,-8.1923)
**1CTP**	(213.8180,115.6160,-64.7866,0.8091,-0.9292,1)	(-0.0009,0.0042,0.0046)	(213.8171,115.6202,-64.7820)
**1ATP**	(3.7499,-7.0372,-2.2543,0.1487,-0.2992,1)	(0.1123,0.0595,0.0011)	(3.8622,-6.9777,-2.2531)
**2AK3(A)**	(86.7048,-71.6582,63.0325,-1.2556,-0.4909,1)	(0.0024,0.0100,0.0080)	(86.7072,-71.6482,63.0405)
1AKE(A)	(133.7850,-177.6240,402.4180,-9.6463,-2.8397,1)	(-0.0046,0.0190,0.0099)	(133.7804,-177.6050,402.4279)
**1GGG(A)**	(-24.8789,-0.3595,-9.9989,-0.0595,0.1928,1)	(-0.0022,-0.0354,0.0067)	(-24.8811,-0.3949,-9.9922)
**1WDN**	(-461.8080,-212.3000,116.8090,-7.9733,17.8943,1)	(0.0085,0.0017,0.0366)	(-461.7995,-212.2983,116.8456)
**2BPG(A)**	(-15.3978,-7.1701,24.6064,2.5494,0.9374,1)	(0.0338,-0.0874,-0.0043)	(-15.3640,-7.2575,24.6021)
1BPD	(2.5596,-4.0506,-1.9642,0.8227,-0.3232,1)	(0.1747,0.1557,-0.0934)	(2.7344,-3.8949,-2.0576)
**1K23(A)**	(51.0180,-42.4661,98.4056,-0.2768,2.2575,1)	(0.0188,0.0056,-0.0073)	(51.0368,-42.4605,98.3983)
**1K20(A)**	(-53.8385,-382.0230,168.5690,-10.6181,-2.5881,1)	(-0.0003,0.0098,0.0221)	(-53.8388,-382.0132,168.5911)
**1URP(C)**	(1.3473,0.5327,0.7839,0.4220,-0.3946,1)	(-0.3103,0.3746,0.2788)	(1.0370,0.9073,1.0626)
2DRI	(56.9057,-23.2339,-54.2220,0.6751,-0.6885,1)	(0.0090,0.0139,0.0035)	(56.9147,-23.2200,-54.2185)

The computational complexity of the entire approach is *O*(*n*^3^), dominated by the calculation of the null space (Mathematica). In practice, though, the linear-time sample generation program (written in Java) is the most time consuming step; depending on the number of clusters in the model, processing time ranged from less than 30 minutes up to 5 hours on a MacBook Pro with a 2.6 GHz Intel Core i7 processor and 8GB of memory. However, the focus of this study was not on execution time; future analysis will rely on an optimized codebase and precise timing experiments.

### Case studies

We now present detailed studies of our analysis on three proteins: calmodulin, the Lysine-Arginine-Ornithine (LAO) binding protein and the Bence-Jones protein. We chose these proteins as they are well-documented in the literature as undergoing conformational changes through hinge-like motions. Studies using NMR [19], x-ray crystallography [20], MD simulation studies [21] and algorithms that combine information from normal modes, experimental thermal factors, bond constraint networks, energetics, and sequence [22], all agree on the mechanism and measurement for hinge motions in calmodulin. The motions of both the LAO binding and Bence-Jones proteins were studied in [23], and the structure of the LAO crystal analyzed in detail in [24].

#### Calmodulin

Calmodulin is a multifunctional, calcium-binding, intermediate messenger protein, which is expressed in all eukaryotic cells. Metabolism, apoptosis, muscle contraction and memory are only few of the many crucial processes mediated by the protein [25]. Calmodulin contains 4 calcium binding sites, with a pair in each of the EF-hand globular domains found at the N- and C-termini; these are connected by a helix with a "weak" center around residue 78. This helix plays a key role in conformational changes in calmodulin: (1) tightening when binding calcium, and (2) unraveling for subsequent peptide binding. We analyze both conformational changes and, as we discuss below, the predicted axes of motions agree, computed to be roughly in the same direction as this helix.

**Figure 6:**
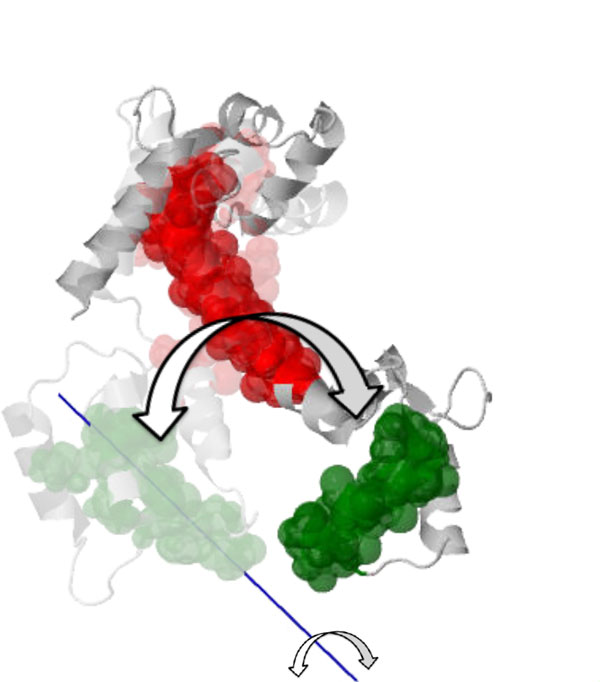
**The *open *state of Ca^2+^-bound calmodulin [PDB:1CLL] is a result of a conformational change from calcium-free calmodulin ([PDB:1CFD], shown faded)**. The two "ends" of the "dumbbell" rotate relative to each other about the "handle" (central alpha helix); the computed axis of motion (blue) of the moving cluster (green) relative to the pinned cluster (red) is consistent with this motion.

**Figure 7:**
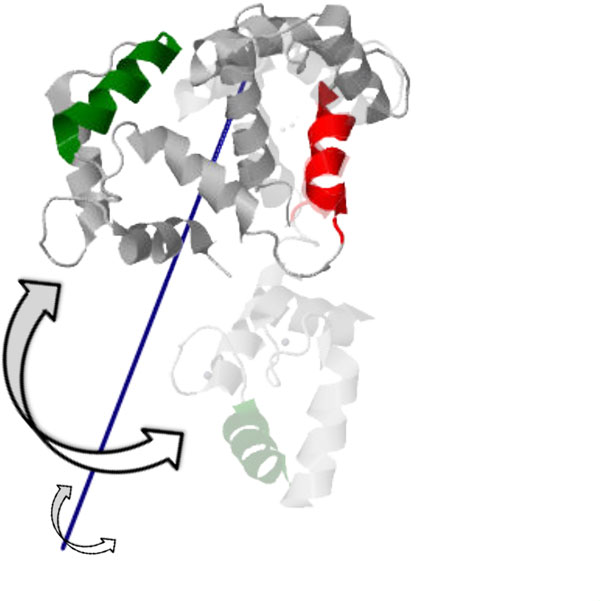
**The *closed *state of Ca^2+^-bound calmodulin [PDB:2BBM] is obtained by another conformational change when a peptide is bound to the *open *state ([PDB:1CLL], shown faded), causing the two globular domains to "wrap" around the peptide (not shown)**. The axis of motion (blue) of the moving cluster (green) relative to the pinned cluster (red) is computed on the *closed *state and is consistent with the motion.

The first conformational change is triggered when calcium binds to calcium-free (apo) calmodulin and causes the central helix to straighten, correlated with a relative rotation of the two globular domains. The resulting conformation is Ca^2+^-bound calmodulin in its *open *state [PDB:1CLL], depicted in Figure 6. We ran our analysis by choosing to pin Cluster 0 (red), computing the relative axis of motion for Cluster 1 (green, containing residues 100-115, and chosen to represent the globular domain at the C-terminus); the blue line depicts the average axis of motion. The calcium-free structure [PDB:1CFD] is shown faded, with the two structures aligned on the central helix residues (65-77) of the pinned cluster (shown in red). Motion of the moving green cluster rotating about the computed axis is consistent with the conformational change; our analysis assigns a twist purity value of about 95, indicating a motion that is almost a pure rotation. A subsequent conformational change occurs when *open *Ca^2+^-bound calmodulin binds a peptide; the two globular domains "wrap" around the peptide, leading to the *closed *state [PDB:2BBM] shown in Figure 7. We pin Cluster 4 (red, containing residues 64-75) and compute the relative motion of Cluster 7 (green, containing residues 101-113) to be consistent with the previous analysis. The axis of motion (blue line) is consistent with a twisting motion that would correspond to this "wrapping" motion and unraveling of the central helix. Indeed, our analysis assigns a twist purity value of around 150, indicating a more general "screw" motion; the *open *conformation [PDB:1CLL] is shown faded, aligned along residues 64-75.

#### LAO binding protein

**Figure 8:**
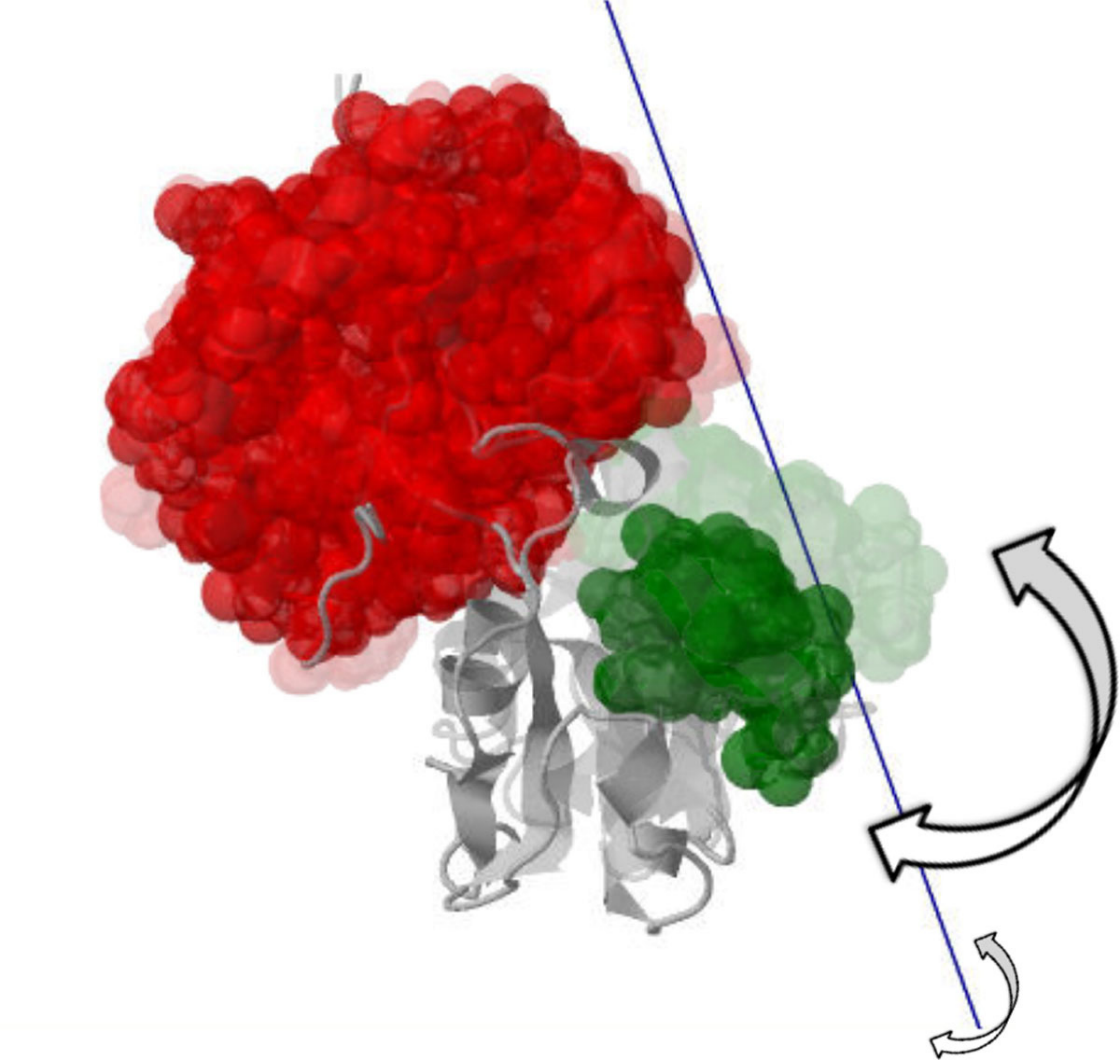
**One lobe of the *open *conformation of the LAO binding protein [PDB:2LAO] is known to rotate relative to the other lobe, resulting in the *closed *conformation ([PDB:1LST], shown faded)**. The computed axis of motion (blue) for the moving cluster (green) relative to the pinned cluster (red) is consistent with this motion.

The Lysine-Arginine-Ornithine (LAO) binding protein is a bacterial peri-plasmic protein that assists the arginine transport system by interacting with membrane-bound receptors. The *open *[PDB:2LAO] and *closed *[PDB:1LST] structures, determined by x-ray crystallography, indicate a rotation of two "lobes" relative to each other [24]. We show the results of our analysis on the *open *structure in Figure 8; Cluster 0 (red) is pinned, and the average axis of motion computed for Cluster 1 (green) is shown in blue. The *closed *structure is shown faded, aligned on residues 1-88; this computed axis is consistent with the rotation, and our analysis computes a twist purity value of around 92.

#### Bence-Jones protein

**Figure 9:**
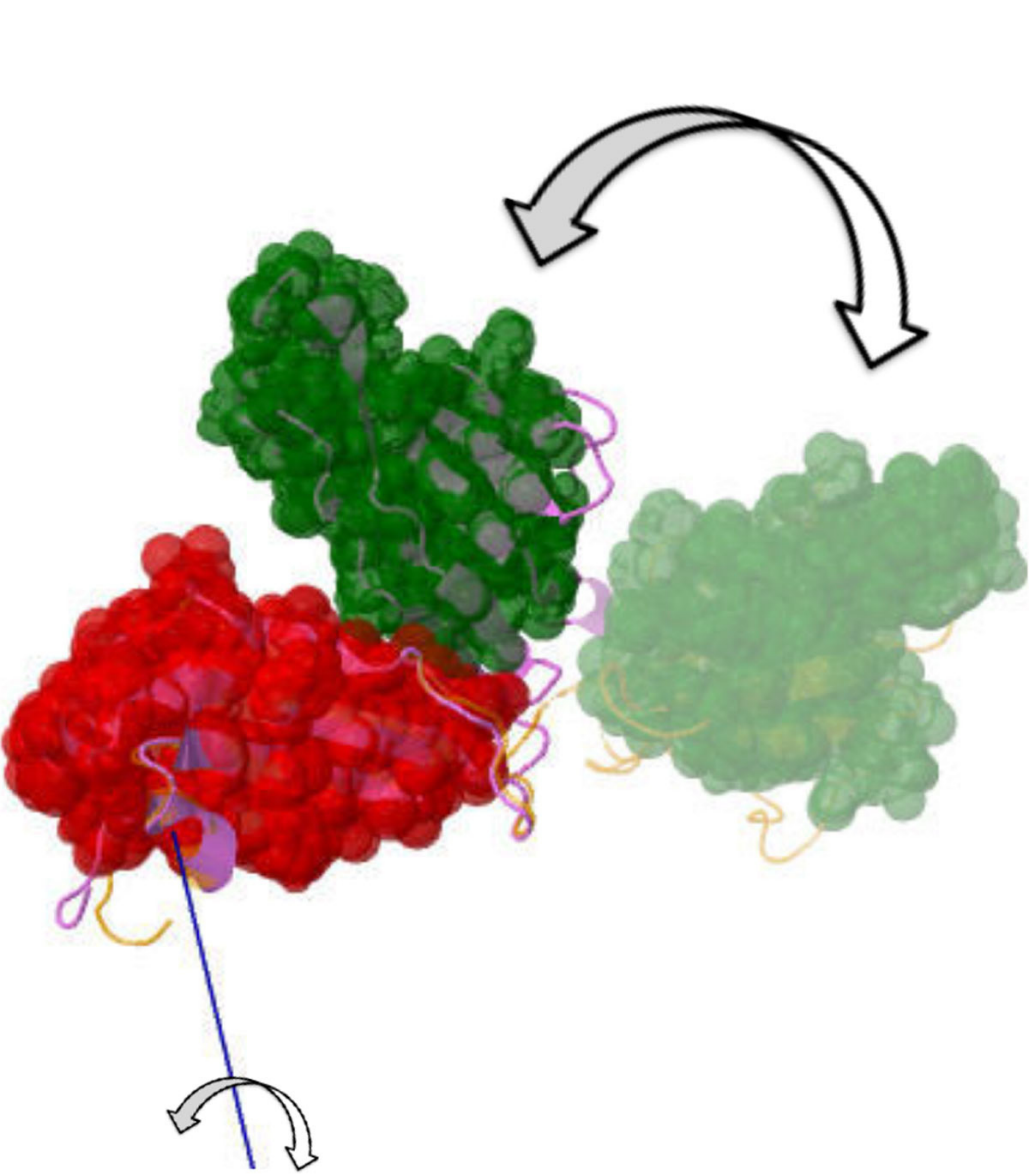
**One subdomain of the Bence-Jones protein rotates relative to the other to move between the *closed *([PDB:4BJL], chain A) and *open *([PDB:4BJL], chain B, shown faded) conformations**. The computed axis of rotation (blue) for the moving cluster (green) relative to the pinned cluster (red) is consistent with this motion.

Bence-Jones proteins are the "light chains of immunoglobins" and "subunits of antibodes" produced by neoplastic (early stage tumor) white blood cells. The presence of the Bence-Jones protein in urine is often an indication of multiple myeloma or bone marrow cancer [26]. Different conformations, determined by ray crystallography, indicate a rotation of one subdomain relative to the other [27]. We show our analysis of the *closed *conformation ([PDB:4BJL], chain A) in Figure 9. We pin Cluster 0 (red) and compute the average axis of motion (blue) of Cluster 1 (green); our analysis computes a twist purity value of around 99, indicating a rotation about the axis consistent with the *open *conformation ([PDB:4BJL], chain B, shown faded and aligned on residues 114-216).

### Axis of motion analysis

Our results demonstrate the potential that rigidity-theoretic analysis has for predicting protein motion, establishing the initial groundwork for future studies. While the use of Jmol to visually validate the predicted axis is intuitive, it can be subjective and highlights the need for a robust computational method that quantifies the validity of the computed data.

**Figure 10:**
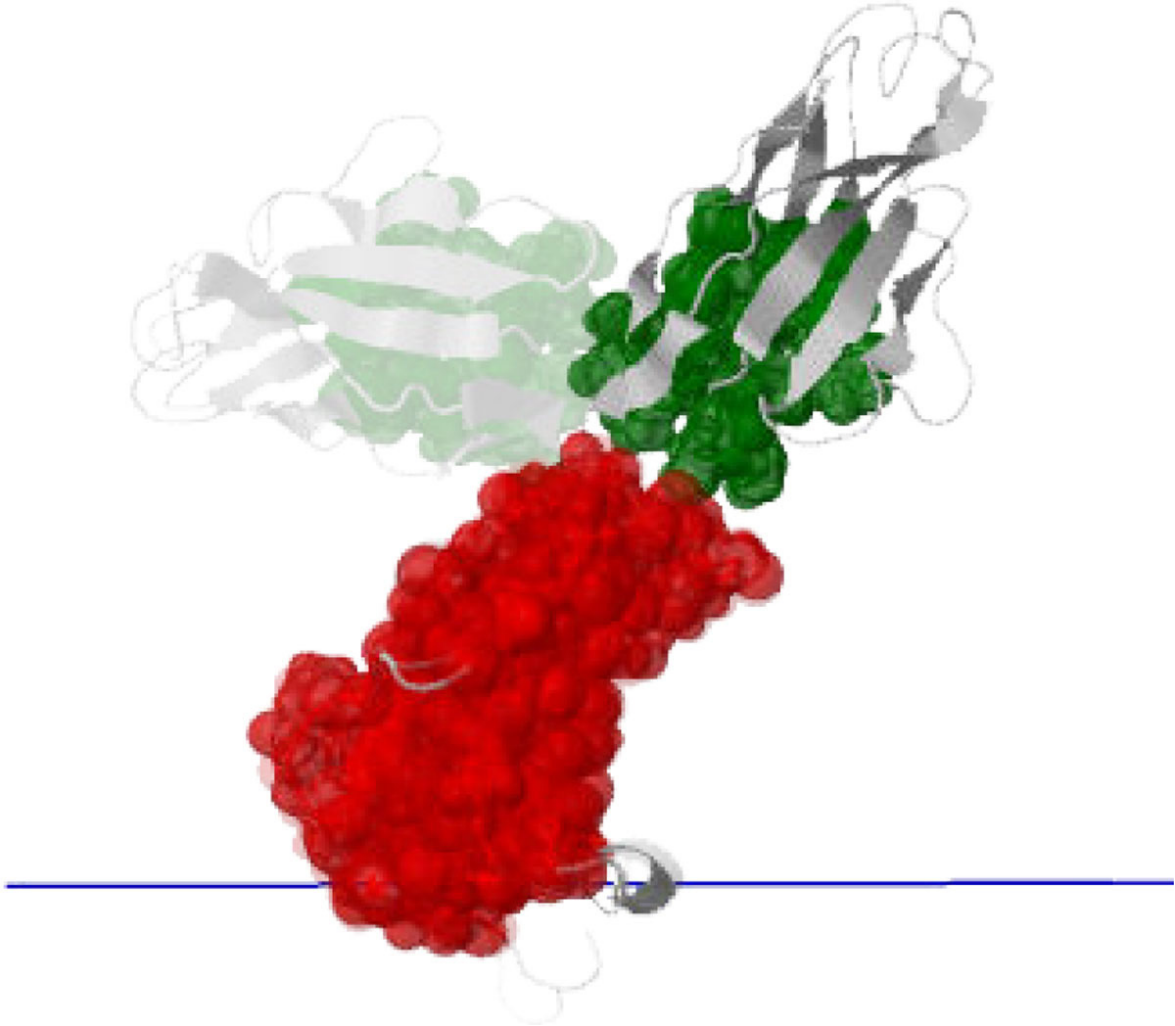
**One subdomain of the Bence-Jones protein rotates relative to the other to move between the *closed *([PDB:4BJL], chain B) and *open *([PDB:4BJL], chain A, shown faded) conformations**. The computed axis of rotation (blue) for the moving cluster (green) relative to the pinned cluster (red) is not consistent with an expected pathway of motion.

**Figure 11:**
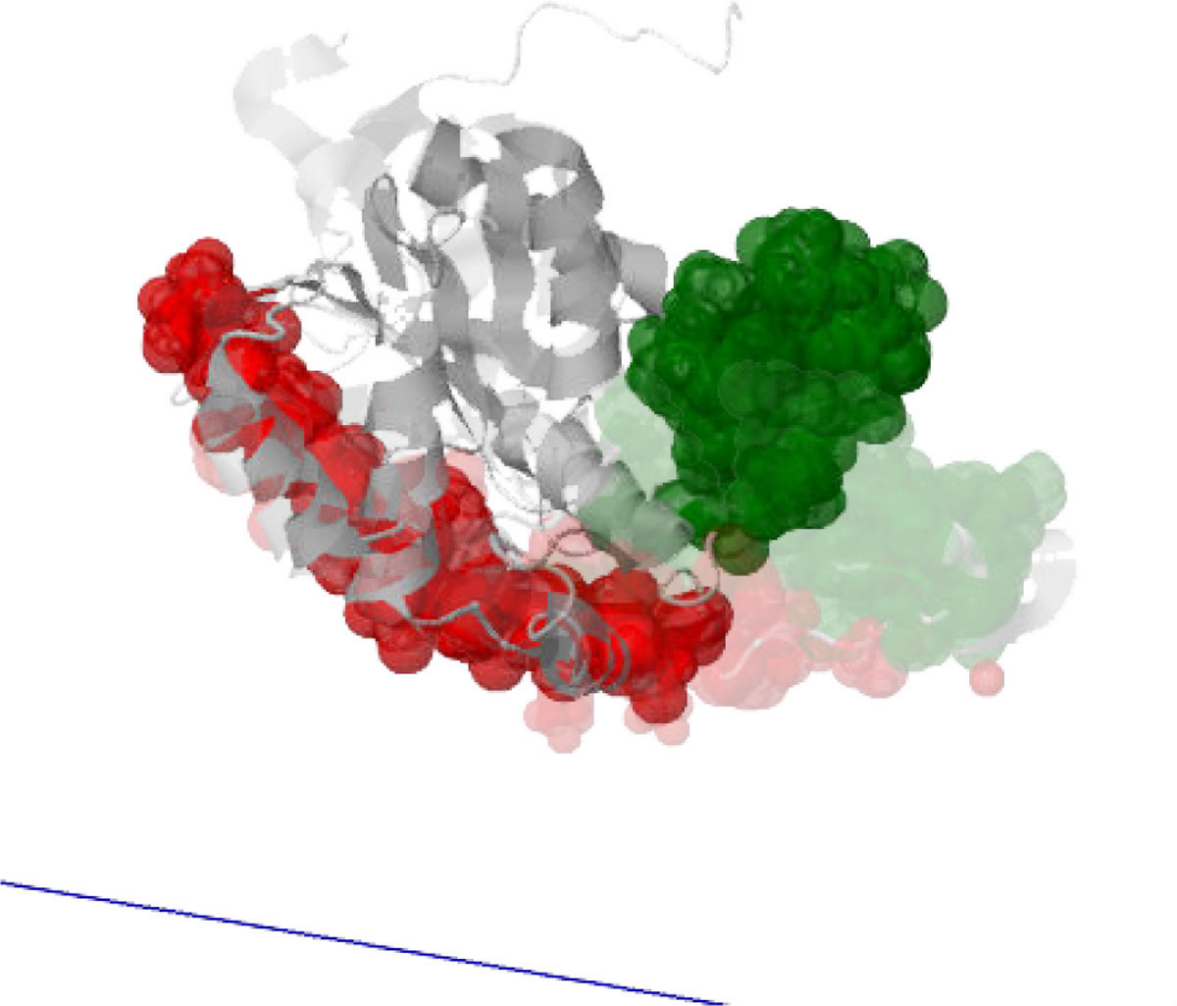
**One subdomain of adenylate kinase rotates relative to the other to move between the *closed *([PDB:1AKE], chain A) and *open *([PDB:2AK3], chain A, shown faded) conformations**. The computed axis of rotation (blue) for the moving cluster (green) relative to the pinned cluster (red) is not consistent with an expected pathway of motion.

**Figure 12:**
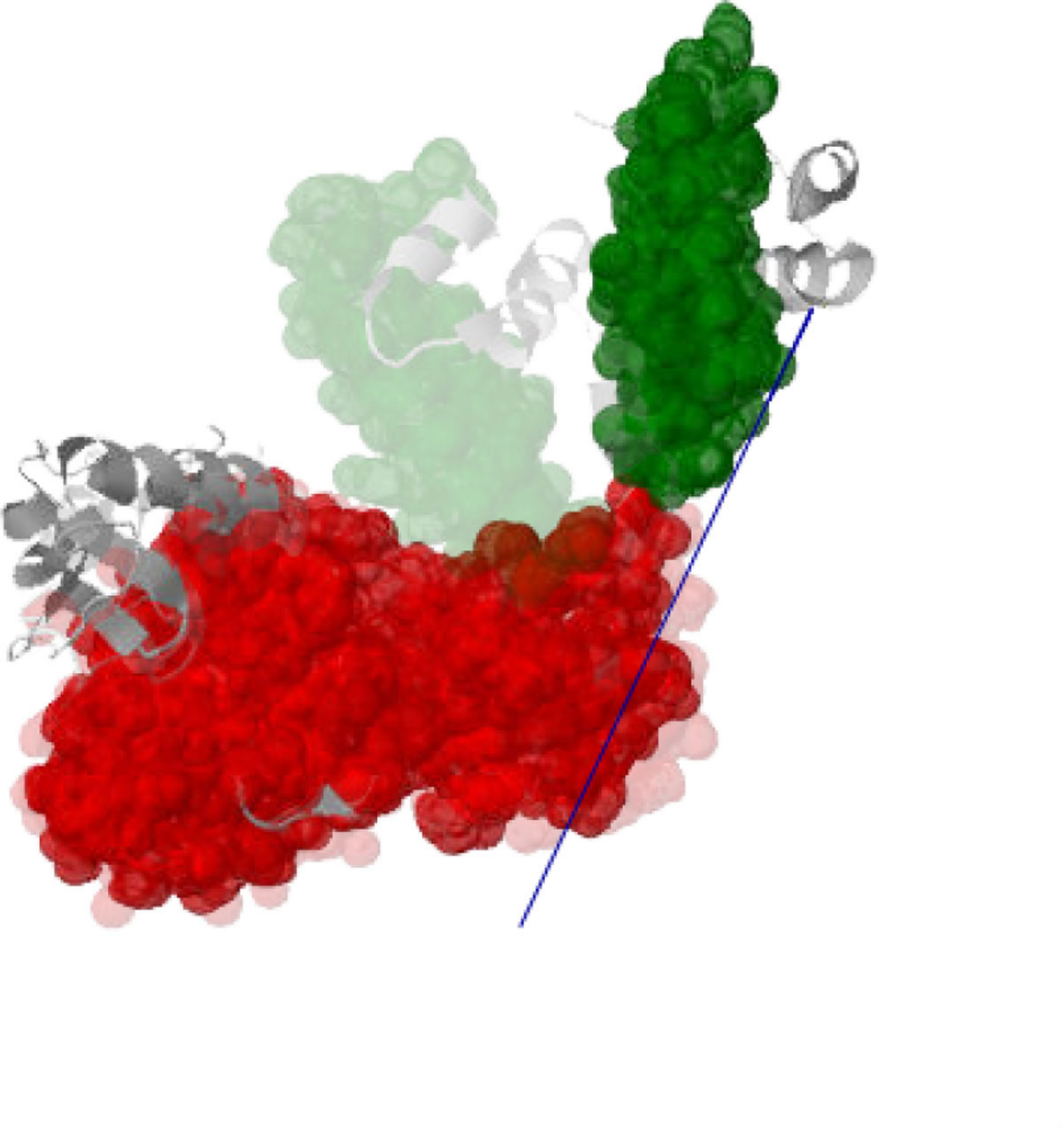
**One subdomain of DNA polymerase *β *rotates relative to the other to move between the *closed *([PDB:1BPD]) and *open *([PDB:2BPG], chain A, shown faded) conformations**. The computed axis of rotation (blue) for the moving cluster (green) relative to the pinned cluster (red) is not consistent with an expected pathway of motion.

**Figure 13:**
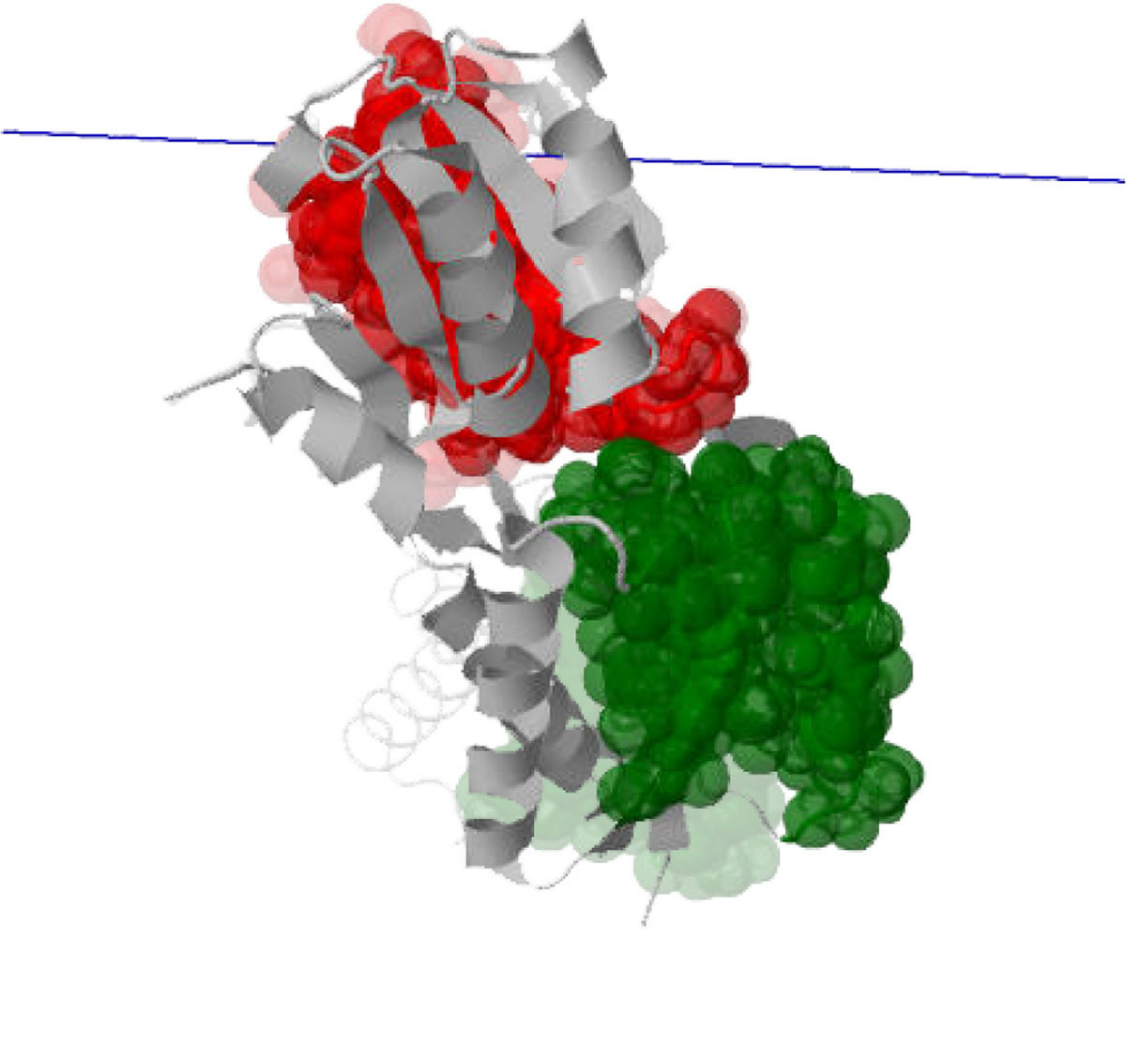
**One subdomain of the ribose binding protein rotates relative to the other to move between the *closed *([PDB:2DRI]) and *open *([PDB:1URP], chain C, shown faded) conformations**. The computed axis of rotation (blue) for the moving cluster (green) relative to the pinned cluster (red) is not consistent with an expected pathway of motion.

For 4 of the structures ([PDB:4BJL,B], [PDB:1AKE,A], [PDB:1BPD] and [PDB:2DRI]), the computed average axis of motion is not visually consistent with the most straightforward movement to a second conformation. Figures 10, 11, 12, 13 depict the pairs of conformations along with the predicted axes of motion; the inconsistencies are more explicit when viewed in 3D.

These structures require further investigation, as the twist data we aggregate over correspond to motions that maintain the geometric modeling of chemical interactions while minimizing steric hindrance. We hypothesize that an inconsistent axis may be due to:

• an infeasible axis produced by the averaging of feasible twists;

• a feasible, but "unexpected" pathway between the two conformations, such as the unfolding of an alpha helix (a potential explanation for the closed conformation of adenylate kinase, Figure 11);

• or, a feasible motion to a conformation that has not been experimentally determined.

## Conclusions

Using rigidity theory, we developed a computational approach for predicting an axis of motion for two domains of a protein, requiring only a **single conformation **as input. We evaluated our approach on a dataset of 19 protein structures, verifying a consistent axis of motion for 15 of them, and presented a detailed discussion of proteins whose motions are well-documented: calmodulin, the LAO binding protein and the Bence-Jones proteins.

Our results show that rigidity theory can be applied to analyze proteins and accurately predict information that may elucidate conformational changes tied to protein function. To the best of our knowledge, calculation of twists from a single conformation has not been done before; however, it would be interesting to compare with standard optimization techniques (such as simulated annealing) by seeking twists whose resulting conformation minimizes energy.

This initial investigation represents the first step to a more comprehensive study. We wish to find the minimum number of samples to generate, as this is the most time-consuming step; a sample set of 100 for calmodulin [PDB:1CFD] seemed to exhibit the same behavior as the sample set of 1000. Since the current approach required close interaction with KINARI to produce an appropriate cluster decomposition, we plan to automate this part of the process in the future, enabling an evaluation of the method on a larger dataset. We ultimately expect to develop a web tool that will allow users to analyze a single structure by uploading or choosing a PDB file. Finally, we seek to develop a computational measure for evaluating the validity of our results instead of visually comparing two conformations using Jmol.

## Structures and figures

All protein structures were obtained from the RCSB Protein Data Bank (using the indicated PDB IDs) [1]. Figures were generated by adapting the output of KINARI [11] and its associated Jmol scripts [28]. Alignment of structures was performed using PyMOL [29].

## Competing interests

The authors declare that they have no competing interests.

## Authors' contributions

RS and MD developed the codebase and performed analysis on an initial set of proteins under the guidance of AL while undergraduates at Mount Holyoke College. SJ and KM provided domain expertise for the hinge protein dataset and subsequent analysis of conformational changes. AL wrote automation scripts and performed experiments on the entire dataset. All authors read and approved the final manuscript.

## Declarations

This article has been published as part of *BMC Bioinformatics *Volume 15 Supplement 8, 2014: Selected articles from the Third IEEE International Conference on Computational Advances in Bio and Medical Sciences (ICCABS 2013): Bioinformatics. The full contents of the supplement are available online at http://www.biomedcentral.com/bmcbioinformatics/supplements/15/S8.
